# Fair inclusion of pregnant women in clinical trials: an integrated scientific and ethical approach

**DOI:** 10.1186/s13063-017-2402-9

**Published:** 2018-01-29

**Authors:** Rieke van der Graaf, Indira S. E. van der Zande, Hester M. den Ruijter, Martijn A. Oudijk, Johannes J. M. van Delden, Katrien Oude Rengerink, Rolf H. H. Groenwold

**Affiliations:** 1 Department of Medical Humanities, Julius Center for Health Sciences and Primary Care, University Medical Center Utrecht, PO Box 85500, 3508 GA Utrecht, The Netherlands; 2 Laboratory of Experimental Cardiology, University Medical Center Utrecht, Utrecht, The Netherlands; 3 Department of Obstetrics and Gynaecology, Academic Medical Center, Amsterdam, The Netherlands; 4 Julius Center for Health Sciences and Primary Care, University Medical Center Utrecht, Utrecht, The Netherlands; 5 Department of Epidemiology, Julius Center for Health Sciences and Primary Care, University Medical Center Utrecht, Utrecht, The Netherlands

**Keywords:** Research ethics, Pregnant women, Research methodology, Fair inclusion, Drug trials, Oversampling, Evidence-based research, Justify exclusion, Phase III trials, Corrective justice

## Abstract

**Background:**

Since pregnant women are severely underrepresented in clinical research, many take the position that the exclusion of pregnant women from research must be justified unless there are compelling “scientific reasons” for their exclusion. However, it is questionable whether this approach renders research with pregnant women fair. This paper analyzes and evaluates when research with pregnant women can be considered as fair and what constitutes scientific reasons for exclusion.

**Methods:**

Conceptual ethical and methodological analysis and evaluation of fair inclusion.

**Results:**

Fair inclusion of pregnant women means (1) that pregnant women who are eligible are not excluded solely for being pregnant and (2) that the research interests of pregnant women are prioritized, meaning that they ought to receive substantially more attention. Fairness does not imply that pregnant women should be included in virtually every research project, as including only a few pregnant women in a population consisting only of women will not help to determine the effectiveness and safety of a treatment in pregnant women. Separate trials in pregnant women may be preferable once we assume, or know, that effects of interventions in pregnant women differ from the effects in other subpopulations, or when we assume, or know, that there are no differences. In the latter case, it may be preferable to conduct post-marketing studies or establish registries. If there is no conclusive evidence indicating either differences or equivalence of effects between pregnant and non-pregnant women, yet it seems unlikely that major differences or exact equivalence exist, the inclusion of pregnant women should be sufficient. Depending on the research question, this boils down to representativeness in terms of the proportion of pregnant and non-pregnant women, or to oversampling pregnant women.

**Conclusions:**

Fair inclusion of pregnant women in research implies that separate trials in pregnant women should be promoted. Inclusion of pregnant women has to be realized at the earliest phases of the research process. In addition to researchers and research ethics committees, scientific advisory councils, funders, drug regulatory agencies, pharmaceutical companies, journal editors and others have a joint responsibility to further develop the evidence base for drug use in pregnant women.

## Background

The development of drugs for obstetric and non-obstetric illnesses for pregnant women is a slowly evolving process. Even though more than half of pregnant women take (prescription) medications during pregnancy for both obstetric and non-obstetric indications [1, 2], there has always been a widespread reluctance to include pregnant women in clinical research due to, among other issues, potential harm to the fetus. Although sound data are unfortunately lacking, there are estimates that the total percentage of women who take medications during pregnancy, either prescribed or over-the-counter, may currently be as high as 64–90% [2–5]. Common medications include painkillers, antibiotics, asthma, sleep and anti-nausea medications [6].

If drugs are tested in pregnant women, studies usually concern investigator-initiated studies of long-existing and long-used medications (that were previously approved for non-pregnant conditions) that are now tested for effectiveness during pregnancy and labor, such as a low-dose aspirin to prevent spontaneous preterm labor. The results of these studies seldom lead to registrations for new indications during pregnancy, but at best to evidence for off-label use. Innovative drugs for pregnant women are rarely developed. As refraining from taking medication during pregnancy could also harm the mother and the fetus, in the past decades regulators, bioethicists and researchers seemed to have reached consensus that the inclusion of pregnant women in research should be promoted [7–12]. Extrapolation of data from studies conducted in men and non-pregnant women is often uncertain, as pregnancy alters the way that drugs are metabolized by the body and act on the body in a fashion difficult to predict from the pharmacokinetics and pharmacodynamics of non-pregnant groups [1, 11, 13, 14]. Risk-benefit profiles are likely to differ as well [8]. Gathering conclusive data in order to develop effective treatments for pregnant women with acute or chronic non-obstetric illnesses, as well as innovative medications for obstetric illnesses, therefore, requires research in pregnant women.

The poor evidence base for drug use in pregnancy is widely regarded as unfair [9]. Already in 1993 the Council for International Organizations of Medical Sciences claimed that the exclusion of pregnant women as a class is unjust [12], and in 1994, the Office of Research on Women’s Health (ORWH) of the Department of Health and Human Services (DHHS) in the United States endorsed the view that pregnant women are to be presumed eligible for participation in clinical research and stated that pregnant women ought to be “fairly enrolled” in clinical research. This view was later supported by regulatory agencies (US Food and Drug Administration (FDA) and the European Medicines Agency (EMA)) [15–17], the US Institute of Medicine [18], and by many individual bioethicists. Despite this longstanding consensus on the need to include pregnant women in clinical research, the situation has not significantly changed since 1994. Exclusion of pregnant women from research is still common practice [19, 20]. A recent review demonstrated that between 1960 and 2013 only about 1% of pharmacokinetic clinical trials were conducted for pregnant women, and the ones that were undertaken had a strong focus on acute labor and delivery issues [21]. Not surprisingly, a 2011 study on all medications approved by the FDA from 1980 to 2010 found that 91% of the medications approved for use by adults did not have sufficient data on safety, efficacy and fetal risk of medication taken during pregnancy [22]. At the same time, the number of pregnant women who take medications, as well as the number of medications that these pregnant women take, has increased [6, 21].

Evidently, even after the awareness of “fair enrollment,” pregnant women remain poorly represented. Among the different reasons for the continuous underrepresentation is the problem that guidelines are ambiguous with respect to if, and when, pregnant women should be included in clinical research and what renders their inclusion fair [23–26]. Many scholars and guidelines currently take the position that fairness comes down to the demand to justify the exclusion of pregnant women from research unless there are compelling “scientific reasons” for their exclusion [9, 25, 27, 28]. It is questionable whether this approach to fairness renders research with women fair, since it has now transformed from the one extreme (no inclusion) to having to justify exclusion except when scientific reasons exist. Furthermore, apart from clear-cut cases, such as shown teratogenicity in preclinical studies or unfavorable high risks for the pregnant woman or the fetus, it is unclear what constitutes a scientifically compelling reason to exclude pregnant women. The National Institutes of Health’s *Policy and Guidelines on the Inclusion of Women and Minorities as Subjects in Clinical Research* (2001 amendment) is currently the most elaborated guidance document to clarify this “scientific reason” in relation to clinical research in women [29]. Nevertheless, we will argue below that this document has methodological and ethical shortcomings when applied to *pregnant women*. Therefore, the aim of this paper is to analyze and evaluate when research with pregnant women can be considered as fair and what constitutes scientific reasons for exclusion.

## Methods

We will first perform a conceptual ethical analysis of fair inclusion and then look at fair inclusion from an integrated ethical and methodological perspective by applying the National Institutes of Health (NIH) Policy document to pregnant women.

It is important to note that we assume that scientific- and justice-based reasons are highly integrated and in principle not easy to distinguish. If research is not designed in a scientifically rigorous manner, participants may unnecessarily be exposed to research risks [30]. We will focus primarily on phase III drug research and we assume that a phase III trial is always preceded by sufficient phase I and phase II trials in pregnant women in order to obtain safety and dosing data to be able to expect that the drug is, and will remain, safe enough in pregnant women, and that, therefore, the risk of serious adverse effects is low. We will not touch upon the level of evidence needed to be able to conduct trials in pregnant women, nor on timing of trials in pregnant women and hence also not on trial designs and models that may speed up knowledge generation in this field. Finally, although our paper focuses primarily on the interests of pregnant women, the findings may also be of relevance to other underrepresented groups including breastfeeding women.

## Results

### Conceptual ethical analysis of fair inclusion

Fair inclusion of study participants in research is one of the core principles of human subjects research [30]. Scandals and tragedies in the past have significantly determined the interpretation of fair subject selection. High-risk research with populations that were “readily available,” such as illiterate, marginalized and powerless groups, has taught us that the scientific objectives of a study and not the “compromised” position nor the “ease of manipulation” should determine the choice of the study population [27, 30]. At the same time, sometimes as a result of an attempt to protect those groups that are easy to recruit, they are categorically excluded which has led to substantial gaps in knowledge about the treatment for conditions that affect these frequently excluded or underrepresented groups, such as children and incompetent persons [27]. Pregnant women take an interesting position among these underrepresented groups since they have not been excluded because of their ease of manipulation but because tragedies with medications that have *not b*een studied in pregnant women, particularly thalidomide and diethylstilbestrol (DES), have caused widespread resistance to test medications in this population [31]. However, the response is the same, the scandals have caused underrepresentation and, therefore, exacerbation of knowledge gaps. Therefore, many currently propose to justify exclusion as a way to promote inclusion unless there is a sound scientific reason not to include them.

The demand to justify exclusion of subpopulations is typically grounded in two principles of justice [18, 32]. Sometimes having to justify exclusion is seen as *justice as equity*, meaning that eligible people should be included without regard to age, gender, race, economic status, or ethnicity. Justice as equity applies to the level of individual research projects, meaning that in every research project pregnant women should be treated as equal to other potentially eligible research participants. As a result, some argue that pregnant women should, unless there are scientific and ethical reasons not to do so, be *routinely included* [33, 34]. Fair inclusion may also be regarded as a form of *corrective justice*, meaning that we should prioritize the inclusion of minorities as long as they have been, and continue to be, underrepresented in research. Mastroianni and colleagues argue that “justice may require a policy of preferential treatment toward these specific areas in order to remedy a past injustice and to avoid perpetuating that injustice” [18]. For pregnant women specifically, it has been claimed that “justice supports the dedicated use of public funds to redress the lack of data about treatments during pregnancy” [35]. This second approach to justice may apply to researchers of specific projects and companies applying for marketing authorization of a drug, but may also be directed at an (inter)national level, applying to funding agencies and governments to promote programs that stimulate research that responds to the health needs of pregnant women [18, 32].

Mastroianni and colleagues discern a third approach to fair inclusion, which aims to fairly benefit all people regardless of their sex or gender and class. According to their third approach, a national research agenda must actively promote research in all areas. As we see it, this third approach is a mixture of the two forms of justice that we have just discerned since it implies that there is no a priori reason not to benefit pregnant women who participate in research (equity) and that specific agencies in a society may be designated to ensure that the interests of women are sufficiently promoted (corrective justice). In addition, the third approach focuses on a just distribution of benefits. This aspect has been disregarded in our paper since we primarily discuss inclusion and exclusion.

It is important to note here that factual inclusion of pregnant women will, as is the case for any research group, also be determined by other ethical considerations such as the potential of pregnant women to give voluntary informed consent and whether the risk-benefit ratio of a study is favorable [18]. For example, due to unknown risks, planning a trial in pregnant women and exposing larger numbers of pregnant women would only be warranted if drug dose and drug safety is sufficiently established in the non-pregnant population. However, for the purposes of this paper we have only considered the implications of the fair inclusion requirement as such, assuming that all other relevant ethical principles apply equally [30].

### Fair inclusion of pregnant women from an integrated ethical and methodological perspective

As we argued above, the NIH Policy document seems to be the most elaborated document that discusses the scientific reasons for the exclusion of subgroups. At the same time, although the document focuses on women and minorities, we may over-interpret the document when applying it to pregnant women since the NIH has some specific guidance on the inclusion of pregnant women [36]. Yet, this specific guidance on the inclusion of pregnant women lacks the criteria mentioned in the NIH Policy document on women and minorities [29]. Therefore, we use the insights in the policy document on women and minorities and consider to what extent these insights can identify legitimate scientific reasons for excluding pregnant women from research. Moreover, before we apply the policy document it is important to note that the NIH Revitalization Act that led to the NIH Policy document has been extensively evaluated from an ethical and legal perspective, but less so from a methodological perspective [18]. Thus, our paper is one of the first attempts to evaluate insights that have existed for a long time and to consider to what extent they are applicable to our discussion on scientific reasons *to exclude pregnant women*. The NIH Policy document presents three scenarios in which (non-pregnant) women and minorities should (not) be included in clinical research (Table 1). In an earlier article we have described our position towards inclusion of these subgroups in research [32]. Below we will summarize this position, and elaborate on it by applying the position to the inclusion of pregnant women in phase III drug research in these three scenarios. In particular, we will evaluate what constitutes a “scientific reason” to justify the exclusion of pregnant women.

**Table 1:** Sections of the National Institutes of Health (NIH) Policy document of relevance to the inclusion of pregnant women

*The NIH is mandated by law (Public Health Service Act sec. 492B, 42 U.S.C. sec. 289a-2) to ensure the inclusion of women and minority groups in clinical research*
Inclusion of women and minorities in NIH-sponsored research is mandated by law: “The [Director of NIH] will, subject to subsection (b) of this section, ensure that…women are included as subjects in each project of such research”…, unless the research “(1) is inappropriate with respect to the health of the subjects;(2) is inappropriate with respect to the purpose of the research; or (3) is inappropriate under such other circumstances as the [Director of NIH] may designate”
Section C: Design of clinical trials In the case of any clinical trial in which women or members of minority groups will under subsection (a) of this section be included as subjects, the [Director of NIH] shall ensure that the trial is designed and carried out in a manner sufficient to provide for a valid analysis of whether the variables being studied in the trial affect women or members of minority groups, as the case may be, differently than other subjects in the trial.
NIH Policy
A. Inclusion of Women and Minorities as Subjects in Clinical Research
It is the policy of NIH that women and members of minority groups and their subpopulations must be included in all NIH-funded clinical research, unless a clear and compelling rationale and justification establishes to the satisfaction of the relevant institute/center director that inclusion is inappropriate with respect to the health of the subjects or the purpose of the research. Exclusion under other circumstances may be made by the [Director of NIH] upon the recommendation of an institute/center director based on a compelling rationale and justification. Cost is not an acceptable reason for exclusion except when the study would duplicate data from other sources. Women of childbearing potential should not be routinely excluded from participation in clinical research. This policy applies to research subjects of all ages in all NIH-supported clinical research studies.
The inclusion of women and members of minority groups and their subpopulations must be addressed in developing a research design or contract proposal appropriate to the scientific objectives of the study/contract. The research plan/proposal should describe the composition of the proposed study population in terms of sex/gender and racial/ethnic group, and provide a rationale for selection of such subjects. Such a plan/proposal should contain a description of the proposed outreach programs for recruiting women and minorities as participants.
B. NIH-defined, Phase III Clinical Trials: Planning, Conducting, and Reporting of Analyses for Sex/Gender and Race/Ethnicity Differences
When an NIH-defined, phase III clinical trial is proposed, evidence must be reviewed to show whether or not clinically important sex/gender and race/ethnicity differences in the intervention effect are to be expected. This evidence may include, but is not limited to, data derived from prior animal studies, clinical observations, metabolic studies, genetic studies, pharmacology studies, and observational, natural history, epidemiology and other relevant studies Investigators must consider the following when planning, conducting, analyzing and reporting an NIH-defined, phase III clinical trial. Based on prior studies, one of the three situations below will apply:
*1. Prior studies support the existence of significant differences*
If the data from prior studies strongly support the existence of significant differences of clinical or public health importance in intervention effect based on sex/gender, racial/ethnic, and relevant subpopulation comparisons, the primary question(s) to be addressed by the proposed NIH-defined, phase III clinical trial and the design of that trial must specifically accommodate this. For example, if men and women are thought to respond differently to an intervention, then the phase III clinical trial must be designed to answer two separate primary questions, one for men and the other for women, with adequate sample size for each
*2. Prior studies support no significant differences*
If the data from prior studies strongly support no significant differences of clinical or public health importance in intervention effect based on sex/gender, racial/ethnic and/or relevant subpopulation comparisons, then sex/gender and race/ethnicity will not be required as subject selection criteria. However, the inclusion and analysis of sex/gender and/or racial/ethnic subgroups is still strongly encouraged
*3. Prior studies neither support nor negate significant differences*
If the data from prior studies neither strongly support nor strongly negate the existence of significant differences of clinical or public health importance in intervention effect based on sex/gender, racial/ethnic, and relevant subpopulation comparisons, then the NIH-defined, phase III clinical trial will be required to include sufficient and appropriate entry of sex/gender and racial/ethnic participants, so that valid analysis of the intervention effects can be performed. However, the trial will not be required to provide high statistical power for these comparisons

#### Relevant differences exist (NIH scenario 1)

In this scenario we “know” (meaning that we are very confident) that the (un)intended effects of the intervention differ between non-pregnant women and pregnant women, yet safety (whether it has unwanted side effects) and efficacy are unknown in magnitude. If we are confident that the effects will differ between women who are pregnant and women who are not, one overall effect estimate based on a study population that is a mixture of these two groups will be little informative and applies neither to pregnant nor to non-pregnant women. The estimated overall effect will apply only to a population with a similar distribution of pregnant and non-pregnant women. In such a situation, indeed, the NIH Policy document advises the setting up different trials or to conduct one trial with two objectives (i.e., investigate the effect in pregnant and non-pregnant women separately, but within the same trial). Thus, if, prior to conducting a trial, it is evident that the effects of an intervention will differ between pregnant and non-pregnant women, running a trial in a group of women, a proportion of whom are pregnant, seems futile. Either a trial is conducted in one of these subgroups, or a larger trial is designed, with pre-specified subgroup analyses looking at the effects of the intervention in the two groups of women separately. Estimating a single overall intervention effect, in our case not taking into account the pregnancy status of a women, will in such a case be irrational.

We think that scenario 1 should be the default for clinical research with pregnant women. Because of the limited evidence about safety and efficacy of drugs in pregnant women we typically rather assume than know that differences exist. If we assume rather than know that there are differences, scenario 1 is preferred in order to avoid taking unnecessary risks and instead be on the safe side. At the same time, it does not follow from our default position that separate trials should always automatically be set up in pregnant women, where this is the case for non-pregnant women to whom the NIH Policy document applies. Pregnant women differ from the general population of women in this scenario since research risks may be different and may affect both the pregnant woman as well as the fetus. As such, research in pregnant women may at times be unwarranted due to risk considerations. Moreover, a disadvantageous result of assuming that scenario 1 should be the default position for which separate trials are preferred, is that we will never establish whether our assumed differences are factual.

Including pregnant women in a trial in a scenario-1 situation may be easier said than done. Practically, there may be reasons not to start a separate or larger trial that also includes pregnant women. To illustrate, if researchers have ample experience in studying interventions in non-pregnant women or if the budget is limited, such that a single trial answering two questions is beyond their ability, there may be no incentive to test a drug in pregnant women. Practical reasons for excluding subgroups may sound valid from a political perspective, but considerations of corrective justice should outweigh those reasons. Attention of designated third parties, such as regulators, governmentally funded research bodies and grant organizations will most likely be essential to stimulate the set-up of separate or larger trials. Corrective justice obligations may be relatively easily fulfilled in the NIH situation, which requires the set-up of different trials for women and minorities and, in our case, pregnant women, but other ethical guidelines for human subject research currently lack this requirement.

#### No relevant differences exist (NIH scenario 2)

In scenario 2 we know (meaning that we are very confident) that the effect is equal in pregnant and non-pregnant women. In the case of equal effects between subpopulations, the NIH “encourages” the inclusion of women and minorities. In the case of non-pregnant women encouragement is conceivable, albeit with hesitations. It is not so clear what is meant by encouragement. If we already know that there are no differences then adding more subgroups seems useless and, therefore, harmful since these subgroups are then unnecessarily exposed to research risks. As in scenario I, it may also be the case that there is no conclusive evidence, but that we *assume* that there are no differences. For instance, if a drug only works locally, is not systemic and does not cross the placenta, such as local anesthetics for suturing wounds or local corticosteroids for skin lesions, we may assume that the effect in pregnant women is similar to that observed in non-pregnant women. If we only assume that no relevant differences exist we could theoretically encourage subgroups to participate for instance because, as the report by Mastroianni and colleagues claims, “greater heterogeneity among research subjects may permit the investigator to spot trends that might otherwise be missed, even if the numbers are too small for statistically reliable subgroup analysis” [18]. However, this exploratory approach will imply a trial with minimum social value for the subgroups included. Simply encouraging inclusion without further specifying the hypothesis and the number needed to include may result in exploratory research only. In most cases, another trial will be needed to demonstrate efficacy which implies that more participants will have to be enrolled in research.

At the same time, if results can be extrapolated, one could argue against the inclusion of pregnant women specifically, because if the trial effects of an intervention are already known, including pregnant women would mean unnecessarily exposing fetuses to potential risks. If the effects of a drug have already been well-studied in non-pregnant women and are known to be applicable to pregnant women, we merely expose pregnant women and their fetuses to research risks. Alternatively, we may *assume* that there are no differences. Accordingly, a precautionary action would be to err on the side of caution which may result in an automatic referral to scenario 1. Or, if the data to be gathered are primarily safety-related and if it is not necessary to conduct a trial in pregnant women to demonstrate efficacy, it may be preferable to conduct post-marketing studies, use registries, and establish small registry studies to pick up safety signals [37].

#### It is unclear whether differences exist (NIH scenario 3)

In this scenario it is unclear whether differences exist, which is, due to the vast lack of clinical research in pregnant women, currently the most common situation in practice. Data on drug safety and drug dose range are usually lacking and phase III trials should not – but are in practice initiated – based on incomplete information. As we set out in the introduction, earlier phase trials will be necessary to minimize the risks and optimize the benefits when pregnant women can be included in phase III trials. Given the objections, precaution requires referral back to scenario 1, and hence to assume that there are differences and thus to apply scenario 1. In other words, scenario 3 is the factual default, whereas scenario 1 should be the normative default for research with pregnant women. But, erring on the side of caution thus does not mean automatically halting any study in which pregnant women may face risks and thereby “paralyze” the situation. One should weigh the risks of participating in the trial versus the risks of not treating pregnant women, or treating them based on insufficient information. Instead, assuming differences may actually imply the set-up of separate drug trials for pregnant women.

Another option in scenario 3 can be oversampling if prior studies have been conducted but the differences between pregnant and non-pregnant groups are unclear. To understand what oversampling of pregnant women implies, we first have to scrutinize the sufficiency criterion. In scenario 3, the NIH Policy document recommends the inclusion of a “sufficient” number of participants from a specific subpopulation in order to be able to perform a “valid analysis” of the intervention. However, this sufficiency criterion, as such, does not guide researchers on how many participants of a certain subpopulation should be included. Evidently, adding only one or two pregnant women to a population consisting only of women is not a substantial inclusion and cannot be sufficient. What is sufficient very much depends on the research setting. If intervention effects may differ between subgroups of pregnant and non-pregnant women, an estimated overall effect could still be informative for the whole population, be it that it is only informative for a population with similar proportions of pregnant and non-pregnant women. In that case, sufficiency comes down to representativeness in terms of the proportion of pregnant and non-pregnant women. So, if one aims at estimating an effect for a future population of women of whom, say, 5% are pregnant, including 5% pregnant women in a trial would be sufficient. However, if one is actually interested in estimating to what extent effects differ between pregnant and non-pregnant women, a larger sample size is required. Effectively oversampling pregnant women, leading to, for example, 50% pregnant and 50% non-pregnant women, would probably be much more efficient for a study with such an objective. Hence, whether sufficiency comes down to (representative) proportionality or oversampling depends on the research question.

And yet, oversampling pregnant women for phase III research in scenario 3 may be challenging for several reasons. First, recruitment and retention of pregnant women in trials is difficult due to a variety of reasons. One of the reasons concerns the individual risk perception of researchers, research ethics committees, sponsors and pregnant women themselves which plays an important role in the inclusion of pregnant women. Even if the research intervention poses low risks and may potentially benefit the pregnant women, when researchers perceive a trial to pose more than low risks to their patients they may be reluctant to recruit eligible participants (gatekeeping) and pregnant women may be reluctant to participate [38]. Second, for many drugs used by pregnant women the purpose will often not be to determine differences in efficacy between pregnant women and non-pregnant women but rather to determine aspects such as effectiveness and safety, including birth defects and teratogenicity. For the latter purpose, it is preferable to follow pregnant women over time because some defects may only manifest over the long term. Moreover, irrespective of the sampling approach, trials may be too small to detect important safety signals. Third, even if pregnant women are oversampled in order to make up 50% of the trial participants, trials that aim at estimating *differences* in intervention effects between subgroups usually require a much larger sample size than studies of main effects [39]. Therefore, also in scenario 3, corrective justice is essential and (inter)national and regulatory agencies have to be found which stimulate the conduct of these projects in pregnant women and the establishment of registries.

## Discussion

Fair inclusion of pregnant women means (1) that pregnant women who are eligible are not excluded solely for being pregnant and (2) that the research interests of pregnant women are prioritized, meaning that they ought to receive substantially more attention. The first component of fair inclusion should not be mistaken for routine inclusion in virtually every trial. Fair inclusion has methodological limitations and exclusion can be justified for scientific reasons. We have described three scenarios that outline where scientific considerations should be taken into account. In scenario 1, it is known that intervention effects for pregnant women differ from those for non-pregnant women. We recommend that pregnant women in this scenario should not be included in phase III drug research that consists of non-pregnant women, but to initiate separate trials for pregnant women during phase III or to conduct phase IV and post-marketing studies.

Alternatively, we know that no differences exist (scenario 2), or we are uncertain whether differences exist (scenario 3). In scenario 2, when we know that there are no differences, it may be best to conduct post-marketing studies or to establish registries, such as the pREGnant registry that has been developed by the Netherlands Pharmacovigilance Center Lareb [40]. Also, when we assume rather than know that there are no differences, we should refer back to the default of scenario 1. In scenario 3, when there is no sufficient prior information, which will in most instances be the case, it may be preferable to return to scenario 1 and to conduct separate trials in pregnant women based on scientific and precautionary considerations. If there is prior information but the information does not indicate either differences or no differences, the inclusion of pregnant women should be sufficient, which explicitly should not mean just enrolling only a few pregnant women in a trial. In this scenario, sufficiency boils down to representativeness in terms of the proportion of pregnant and non-pregnant women or to actually oversampling of pregnant women, depending on the actual research question.

Regarding the second component of fair inclusion, our paper has shown that fair inclusion cannot, and should not, be realized at the moment of ethical review of already designed research projects, but rather that fair inclusion requires a joint effort. Due to the current vagueness of the demand to justify exclusion unless scientific reasons exist and the ambiguity as to the level at which and the actors at whom fair inclusion is directed, no group or institution seems to make fair inclusion its sincere priority.

At present, it seems that fair inclusion only comes into play at the moment of ethical review of already designed individual research projects. However, our paper has demonstrated that the establishment of separate trials has to be realized at the earliest phases of research with pregnant women and that the demand to justify the exclusion of pregnant women cannot be bestowed upon individual researchers and research ethics committees, since protocols are not easily adjusted once researchers have planned their study methods and budgets may be restricted. Additionally, researchers that may be willing to include more pregnant women or to develop separate trials will need extra budget to do so. And thus funders and scientific advisory councils must see it as their priority to promote research with pregnant women and to facilitate the research infrastructure [18]. In this respect, it will also be important to pay more attention to in vitro studies, that currently hardly distinguish between sexes in cell lines and hence contribute to the poor pre-clinical evidence base for drugs in (pregnant) women.

Moreover, in order to develop truly innovative medications for pregnant women, we cannot rely on investigator-initiated research only and we have to look at pharmaceutical companies. Pharmaceutical companies may be asked to substantially invest in sex-specific dosage or medications, yet, with the costs involved in research and development on this topic, together with additional packaging, marketing and liability fears, they may, understandably, be reluctant. Their additional risk is that an alternative company will claim equal effectiveness for both men and women for their compound, which may be preferred by physicians and society. The marketing campaign for sex-specific medications could turn out to be detrimental. Nevertheless, this year Ferring Pharmaceuticals launched NOCDURNA with gender-specific doses tailored to men and women. The success of this compound and the success of the gender-specific strategy is to be determined in the coming years.

In addition, the integrated analysis of fair inclusion has demonstrated that in most cases it will be essential to establish separate trials or registries and this is typically an activity that necessitates the involvement of authorities, such as national pharmacovigilance centers or regulatory authorities such as the FDA and EMA. However, although the role of the FDA and EMA is regulatory and they may guide the directions, they cannot require of pharmaceutical companies to conduct separate trials in (pregnant) women, unless it is laid down in a regulation or directive such as the EU regulation, comparable to research with children [41]. Similar to the Paediatric Regulation in Europe with a Paediatric Committee and the requirements for Paediatric Investigation Plans (PIPs) for marketing approval, the EMA could establish a pregnancy committee and require pregnancy investigation plans if the drug can potentially be used by pregnant women.

Additional stakeholder groups are journal editors and pregnant women themselves. Journal editors could for instance require subgroup analyses from researchers who submit papers to their journal. Currently, this requirement is still a rarity and does not apply to the conduct of separate trials. Pregnant women could associate in patient groups which, in other medical fields, such as the field of orphan diseases or pediatric research, has had success in stimulating drug development. Without patient groups, radical breakthroughs can only be initiated by others than those whose interests are at stake.

In sum, although it is beyond the scope of this paper to conclusively state whose responsibility it is to ensure corrective justice and to prioritize the health interests of pregnant women in research, our paper shows that fair inclusion of pregnant women in research must primarily be seen as a joint responsibility to further the evidence base for drug use in pregnant women.

## Conclusions

The demand to justify the exclusion of pregnant women from research is not only essential for reasons of equity but also for reasons of corrective justice. Since scientific knowledge on the effects of treatments for the health needs of pregnant women is relatively underrepresented, fair inclusion implies that intensive stimulation of research in this population is justified. Fairness does not imply that pregnant women should be included in virtually every research project. Inclusion of only a few pregnant women in a population of women will not help to determine the effectiveness and safety of a treatment in pregnant women. If pregnant women are included it should be done representatively or they should be oversampled in order to be able to determine a difference in intervention effects between groups of pregnant and non-pregnant women. In the few cases where we may be certain that there are no differences between pregnant and non-pregnant women, we should conduct post-marketing studies or arrange the establishment of registries. But, since evidence is typically limited for the treatment of health conditions that affect pregnant women, we either know, or otherwise have to assume, that pregnant women differ from other subpopulations. Separate trials may then be preferable. The current vagueness of the demand to justify exclusion unless scientific reasons exist seems to indicate that fair inclusion only comes into play at the moment of ethical review of already-designed individual research projects. However, fair inclusion is not only an obligation for individual researchers and research ethics committees. The development of separate trials has to be realized at the earliest phases of research with pregnant women. In addition to researchers and research ethics committees, scientific advisory councils, funders, drug regulatory agencies, pharmaceutical companies, journal editors and others all have a joint responsibility to further the evidence base for drug use in pregnant women.

## Abbreviations

DES: Diethylstilbestrol
DHHS: Department of Health and Human Services (DHHS)
EMA: European Medicines Agency
FDA: US Food and Drug Administration
NIH: National Institutes of Health
ORWH: Office of Research on Women’s Health
PIPS: Paediatric Investigation Plans
